# Metabolic risk factors for liver inflammation in a cohort of chronic hepatitis C patients

**DOI:** 10.1186/1471-2334-14-S7-O14

**Published:** 2014-10-15

**Authors:** Mihaela Andreea Rădulescu, Victoria Aramă, Daniela Ioana Munteanu, Raluca Ioana Mihăilescu, Cătălin Tilişcan, Cristina Popescu, Viorica Poghirc, Irina Lăpădat, Smaranda Gliga, Sorin Ștefan Aramă, Adrian Streinu-Cercel

**Affiliations:** 1 Carol Davila University of Medicine and Pharmacy, Bucharest, Romania; 2 National Institute for Infectious Diseases "Prof. Dr. Matei Balş", Bucharest, Romania

## Background

Beside the classic risk factors for disease progression in chronic hepatitis C (CHC) patients, metabolic disturbances came to attention in the last decade as important factors influencing disease progression and liver activity. This study aimed to evaluate metabolic factors in relation to liver inflammation in a cohort of CHC patients.

## Methods

We conducted a cross-sectional non-interventional study on CHC patients evaluated in a tertiary hospital in Bucharest between December 2012-August 2013. We measured fasting serum lipids, glucose, liver transaminases, inflammatory proteins, and viral load. We calculated body-mass index (BMI) and waist-to-hip ratio (WTH). Cardiovascular risk was assessed with Framingham risk score, metabolic syndrome was defined with ATPIII criteria. Liver histology was assessed with non-invasive Fibromax tests (Biopredictive, France). For statistical analysis we used SPSS (version 12.0).

## Results

We enrolled 117 CHC patients compared to 30 uninfected controls. Median age was 54 years [45-61], sex ratio was F:M 1.8. Sixty-one patients (52.1%) had low-grade activity score (A0-1) and 56 (47.8%) had important liver activity (A2-3). In univariate analysis liver activity score (ActiScore) was correlated to age (Spearman rho=0.353, p<0.001). ActiScore was correlated to FibroScore, SteatoScore and NashScore (Spearman rho=0.770, 0.528, 0.439, p<0.001 for all comparisons). Also ActiScore was correlated to viral load (rho=0.262, p=0.023). Regarding metabolic factors, ActiScore was positively correlated to waist circumference and WTH ratio (rho=0.200, p=0.034, respectively rho=0.290, p=0.002) and inversely correlated to serum cholesterol and LDL (rho=-0.262 and -0.294, p=0.005 for both). Also ActiScore was negatively correlated to C-reactive protein and serum fibrinogen (rho=-0.318, p=0.001 and rho=-0.340, p<0.001).

The number of criteria for metabolic syndrome and Framingham risk score were correlated to ActiScore (rho=0.211, p=0.034, respectively rho=0.510, p<0.001). When we compared low activity (A0-1) to high activity (A2-3) patients in logistic regression, risk factors for high activity were age (OR 1.07), SteatoScore (OR 3.93) and NashScore (OR 3.79, p<0.001 for all variables), while serum cholesterol, C-reactive protein and fibrinogen were protective factors (OR 0.042, 0.062, 0.071, p=0.004, 0.025 and 0.008).

## Conclusion

In CHC patients’ metabolic factors play an important role in disease activity. Liver metabolic disease had a negative predictive role, while serum cholesterol and inflammatory markers seemed to have a protective role against liver inflammation.

